# „Verankerung“ von Klassifikationskriterien für axiale Spondyloarthritis – wie weit ist gut für die Diagnosestellung?

**DOI:** 10.1007/s00393-020-00752-z

**Published:** 2020-02-10

**Authors:** J. Braun, U. Kiltz, X. Baraliakos

**Affiliations:** Rheumazentrum Ruhrgebiet und Ruhr, Universität Bochum, Claudiusstr. 45, 44649 Herne, Deutschland

Die Autoren der Studie haben einen interessanten Beitrag zum besseren Verständnis der relativ neuen Entität axiale Spondyloarthritis (axSpA) geleistet, indem sie eine große Zahl deutscher Rheumatologen zu dem Thema befragt haben [1]. Auch wenn sich nur ein relativ kleiner Teil rückgemeldet hat, haben insgesamt doch mehr als 100 Rheumatologen den Fragebogen beantwortet. Die Arbeit bezieht sich auf die Akzeptanz und Rezeption der ASAS-Klassifikationskriterien für axSpA [2] in Deutschland. Die Kriterien grenzen den Kreis derer, die als axSpA klassifiziert werden können, dadurch ein, dass deren Rückenschmerzen schon 3 Monate bestanden und vor dem 45. Lebensjahr begonnen haben müssen. In den letzten Jahren ist häufig darauf hingewiesen worden, dass Klassifikationskriterien nicht für die Diagnosestellung verwendet werden sollen, zuletzt in der eben erschienenen S3-Leitlinie [3].

Obwohl das von den Autoren ausdrücklich ebenso gesehen wird, ist die Fragestellung der Studie doch, inwieweit und in welchem Maß die Klassifikationskriterien akzeptiert und angewendet werden. Das erscheint widersprüchlich, denn Klassifikationskriterien sind ja eben für klinische Studien (Klassifikation) und nicht für individuelle Patienten (Diagnose) geschaffen. Einige Beispiele sind vor einigen Jahren publiziert worden [4].

Es ist erfreulich, dass nahezu alle Rheumatologen die ASAS-Klassifikationskriterien für axSpA kennen und 90 % sie als Fortschritt ansehen, aber die Information, dass über 80 % die Kriterien im Alltag anwenden, erscheint aus den genannten Gründen problematisch, denn das trägt sehr wahrscheinlich zu einer nicht zu vernachlässigenden Anzahl von Fehldiagnosen bei – so wie es die gemessene Sensitivität und Spezifität der Kriterien dokumentiert [2] und wie es die letzten Ergebnisse von MRT-Studien auf Bevölkerungsebene [5] eindrucksvoll demonstriert haben. Diese Problematik wird von den Autoren im Übrigen ähnlich gesehen [1]. Nichtsdestoweniger sind es für Klassifikationskriterien natürlich dieselben klinischen Symptome und Befunde, aus denen sich letztlich auch die Diagnose zusammensetzt, aber sie werden eben vom Facharzt bzw. Experten bewertet und gewichtet – und eben keine Kästchen angekreuzt. Ein von ASAS diagnostischer Algorithmus [6] ist hierfür aber allenfalls partiell hilfreich. In diesem Zusammenhang hat die Mehrheit der befragten Rheumatologen erfreulicherweise der Aussage zugestimmt, dass die Gefahr besteht, dass axSpA-Patienten mit TNF-Inhibitoren behandelt werden, bei denen sich die Diagnose im Verlauf dann als falsch erweist [1].

Der andere damit verbundene Punkt, den wir angesichts dieses wichtigen Themas ansprechen möchten, ist die Unterscheidung zwischen röntgenologischer (r-axSpA) und nicht-röntgenologischer axSpA (nr-axSpA). Dies ist von Bedeutung, weil diese Unterscheidung auf der einen Seite wegen der unterschiedlichen Zulassungen für r‑axSpA und nr-axSpA formal wichtig ist. Auf der anderen Seite ist es aber offensichtlich, dass die Unterscheidung zwischen diesen beiden Entitäten wegen der bestehenden Unsicherheit in der Beurteilung von konventionellen Röntgenbildern der Sakroiliakalgelenke (SIG) mit begrenzten strukturellen Veränderungen (Grad 1 und 2) unscharf und schlecht reproduzierbar ist [7]. Auf der anderen Seite ist die Krankheitslast wenig unterschiedlich [8]. Daraus resultiert die inzwischen international weitverbreitete Ansicht, dass sich der Terminus nr-axSpA nicht als Diagnose, sondern nur als Klassifikation eignet [9]. Das bedeutet, dass die Diagnose von entsprechenden Patienten einfach nur axSpA lauten sollte. Sonst würde ja auch die etwas merkwürdige Konstellation möglich sein, dass ein Patient mit mehreren Syndesmophyten, aber keinen sicheren SIG-Veränderungen als nr-axSpA bezeichnet werden könnte. Der Terminus ankylosierende Spondylitis (AS) muss unter Umständen allerdings bei gutachterlichen Fragen in bestimmten Fällen weiter genutzt werden. Infliximab ist im Übrigen der einzige TNF-Blocker, der nur für die Indikation AS und nicht für nr-axSpA zugelassen ist. Für die IL-17-Inhibitoren ist eine Zulassung für nr-axSpA bald zu erwarten. Abschließend sei darauf hingewiesen, dass die Diagnosekodierung mit dem ICD-10 Code M45.09 beide Entitäten einschließt, dies ist für ICD-11 anders geplant.

Für die Einschätzung der unterschiedlichen Zuordnung zu nr-axSpA bzw. r‑axSpA sollte man sich darüber im Klaren sein, dass nach 2 Jahren Symptomdauer (entzündlicher Rückenschmerz) schon mehr als 20 % der Patienten definitive strukturelle Veränderungen in den SIG aufweisen [10] – d. h. dass diese Patienten in den meisten nr-axSpA-Studien (mit längerer Symptomdauer bei Einschluss) gar nicht mehr in dieser Kategorie auftauchen.

Nicht zu vernachlässigen sind auch der zunehmend klare Unterschied zwischen männlichen und weiblichen axSpA-Patienten [11, 12] sowie die relativ große Häufigkeit von anderen Ursachen für Rückenschmerzen bei Patienten mit axSpA [13, 14], dies haben wir vor Kurzem im Hinblick auf die Differenzialdiagnose detailliert erörtert [15].

Abschließend sei nochmals betont, dass die Arbeit der Kollegen aus Halle angesichts der Komplexität der Problematik Klassifikation – Diagnose unseres Erachtens einen sehr wichtigen Beitrag für das Gesamtverständnis der Erkrankung axSpA darstellt.
